# Phosphorylation of Akt by SC79 Prevents Iron Accumulation and Ameliorates Early Brain Injury in a Model of Experimental Subarachnoid Hemorrhage

**DOI:** 10.3390/molecules21030325

**Published:** 2016-03-10

**Authors:** Shuangying Hao, Chuanhui Song, Longcheng Shang, Yu Jiang, Tong Qiao, Kuanyu Li

**Affiliations:** 1 Jiangsu Key Laboratory for Molecular Medicine, Medical School of Nanjing University, Nanjing 210093, China; 2 Department of Vascular Surgery, the Affiliated Drum Tower Hospital of Nanjing University Medical School, Nanjing 210008, China

**Keywords:** subarachnoid hemorrhage, SC79, Akt phosphorylation, oxidative stress, iron homeostasis, Fe-S cluster biogenesis

## Abstract

Previous studies have demonstrated that activation of Akt may alleviate early brain injury (EBI) following subarachnoid hemorrhage (SAH). This study is undertaken to determine whether iron metabolism is involved in the beneficial effect of Akt activation after SAH. Therefore, we used a novel molecule, SC79, to activate Akt in an experimental Sprague–Dawley rat model of SAH. Rats were randomly divided into four groups as follows: sham, SAH, SAH + vehicle, SAH + SC79. The results confirmed that SC79 effectively enhanced the defense against oxidative stress and alleviated EBI in the temporal lobe after SAH. Interestingly, we found that phosphorylation of Akt by SC79 reduced cell surface transferrin receptor-mediated iron uptake and promoted ferroportin-mediated iron transport after SAH. As a result, SC79 administration diminished the iron content in the brain tissue. Moreover, the impaired Fe-S cluster biogenesis was recovered and loss of the activities of the Fe-S cluster-containing enzymes were regained, indicating that injured mitochondrial functions are restored to healthy levels. These findings suggest that disrupted iron homeostasis could contribute to EBI and Akt activation may regulate iron metabolism to relieve iron toxicity, further protecting neurons from EBI after SAH.

## 1. Introduction

Stroke is a major cause of morbidity and mortality worldwide. Although subarachnoid hemorrhage (SAH) accounts for only 5% of all strokes, it often leads to high mortality [1]. Early brain injury (EBI) is an initial pathological process within the first 72 h of acute cerebral vascular events, plays a pivotal role in disability and death after SAH [2]. The pathogenesis of EBI resulted from various mechanisms, including oxidative stress and inflammation after SAH [2].

Previous studies have indicated that iron deposition is involved in the pathogenesis of EBI after experimental SAH [3] and the iron chelator deferoxamine (DFO) may attenuate DNA damage, lessen induction of iron-handling proteins, and reduce mortality [4]. Cellular iron is mainly taken up via the internalization of the complex of iron bound transferrin-transferrin receptor. An excess of iron may participate in the formation of the highly-reactive hydroxyl radical via Fenton reaction to further result in oxidative damage of cells. Published reports have indicated that exposure of murine fibroblasts to peroxide enhances the expression of transferrin receptor (TfR) and promotes iron uptake [5]. Peroxide-induced dichlorodihydrofluorescein (DCFH) oxidation is catalyzed by iron, which is transported through TfR in endothelial cells, and antioxidants can inhibit TfR expression and iron uptake [6]. Thus, oxidative stress and iron signaling may synergistically amplify a vicious cycle. Therefore, inhibiting reactive oxidative species (ROS) production or/and increasing ROS scavenger may protect cell against ROS-induced iron overload.

The mechanism that activation of Akt enhances the defense against oxidative stress is revealed that a region of *SOD1* (encoding Cu, Zn-superoxide dismutase) promoter between −552 and −355 was targeted by PI3K and phosphorylated Akt [7]. Activation of Akt resulted in higher mRNA and protein levels of SOD1 and lower ROS levels [7]. Another study has also indicated that antioxidant butin attenuates oxidative stress by activating Nrf2-mediated SOD2 induction via the PI3K/Akt signaling pathway [8]. The acute activation of Akt in the brain confers protection against EBI [9] and deactivation of Akt contributes to pathogenesis of EBI [10]. Despite many promising therapeutics have shown the beneficial effects of Akt activation in animal models of SAH, most elevating Akt signaling drugs are unsuccessful for clinically effective therapies, which is likely due to the lack of specificity for Akt activation. SC79, a novel Akt activator described by Jo *et al.* [11], binds specifically to the PH domain of Akt to cause a conformational change that favors its activation. Excitingly, their results have demonstrated that SC79 suppresses excitotoxicity and alleviates stroke-induced neuronal death. Importantly, SC79 is cell-permeable without any side-effects reported, indicating that the molecule could possibly be used as a novel drug in patients. In view of the above facts, we evaluated whether iron metabolism is involved in the beneficial effect of Akt activation after SAH.

## 2. Results

### 2.1. SC79 Activates Akt and Protects Cells against Oxidative Stress after SAH

**Figure 1:**
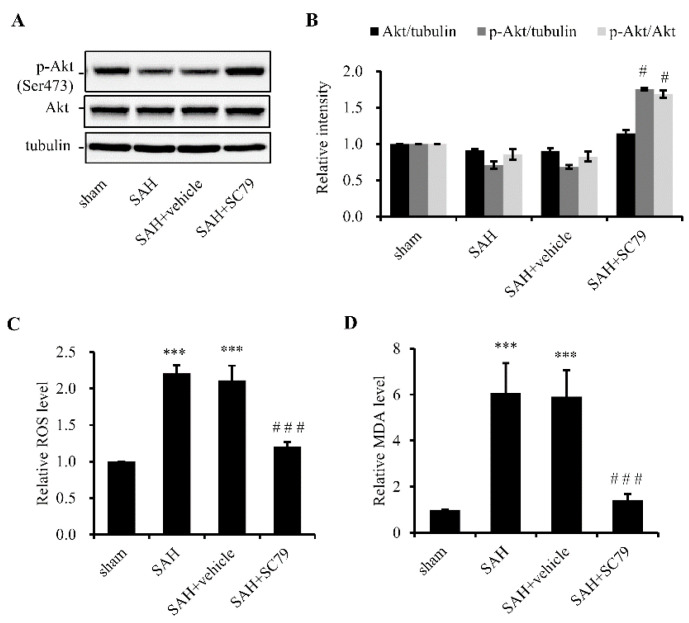
Akt phosphorylation by SC79 administration protects cells against oxidative stress after SAH.Arterial blood (300 μL, normal saline in the sham group) was injected into the prechiasmatic cistern of rats. DMSO (5 μL/rat) and SC79 (100 μg/rat) were given intracerbroventricularly (ICV) at 30 min after SAH as SAH + vehicle and SAH + SC79 groups, respectively. The temporal cortex was harvested 24 h after SAH. (**A**) SC79 activation of Akt by SC79, revealed with Western blotting. The left panel shows a representative result of the levels of total (Akt) and phosphorylated (p-Akt, Ser473) Akt. The right panel shows the quantitative data. Tubulin was used as a loading control; (**B**,**C**) show alleviation of oxidative stress by SC79 treatment. ROS production (**B**) and MDA levels (**C**) were shown. Data are expressed as mean ± SD (*n* = 8 in each group). *** *p* < 0.001 *vs.* the sham group, # *p* < 0.05 and ### *p* < 0.001 *vs.* the SAH group. SAH, subarachnoid hemorrhage; ROS, reactive oxidative species; MDA, malondialdehyde.

To determine the capability of SC79 to induce Akt activation, 100 µg of SC79 were ICV-administered 30 min post SAH. The rats were sacrificed 24 h later, and Western blot analysis was performed to examine the level of p-Akt with the temporal lobe samples. Our result confirmed that SC79 administration increased the level of p-Akt without any obvious changes of the total Akt level (Figure 1A). SAH can induce ROS production and increase the lipid peroxidation; therefore, we examined the levels of ROS and MDA. The production of both ROS and MDA significantly increased after SAH, which was markedly reduced by treatment with SC79 (Figure 1B,C). These data are consistent with the previous reports [7,8,9,10,12], in which activation of Akt reduces the challenge of oxidative stress.

### 2.2. SC79 Administration Mitigates SAH-Induced Iron Accumulation

**Figure 2:**
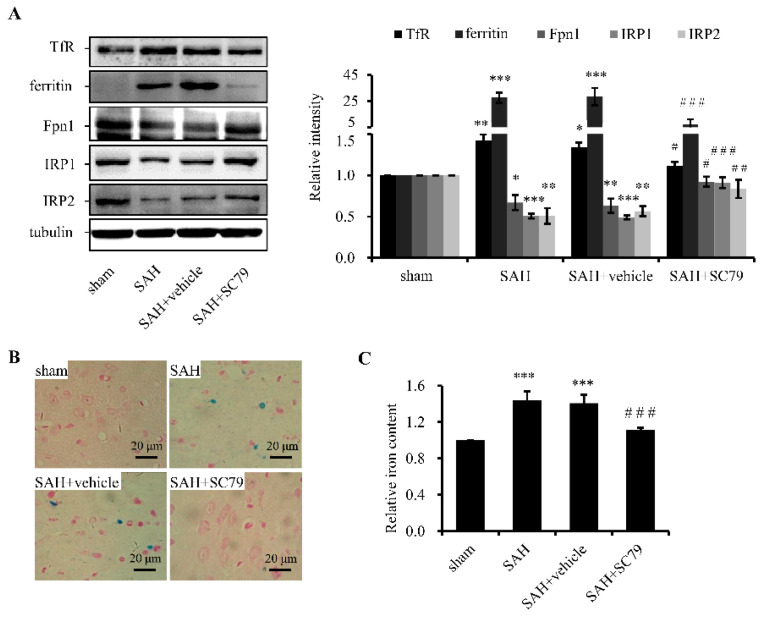
SC79 administration after SAH improves the disrupted iron homeostasis. (**A**) Western blotting for the protein levels of TfR, ferritin, Fpn1, IRP1, and IRP2 of the rat temporal cortex. Left panel: a representative protein levels of TfR, ferritin, Fpn1, IRP1, and IRP2. Right panel: quantitative data of the protein levels. Tubulin was detected as a loading control; (**B**) Prussian blue iron staining; and (**C**) iron content determined by ferrozine assays. Data are expressed as mean ± SD (*n* = 8 in each group). * *p* < 0.05, ** *p* < 0.01, *** *p* < 0.001 *vs.* the sham group, # *p* < 0.05, ## *p* < 0.01, ### *p* < 0.001 *vs.* the SAH group. Definition of sham, SAH, SAH + vehicle, and SAH + SC79 groups is the same as in Figure 1. Fpn1, ferroportin 1; TfR, transferrin receptor, IRP1/2, iron regulatory protein 1/2.

Cellular iron homeostasis is tightly regulated by TfR for iron uptake, ferritin for iron storage, and ferroportin (Fpn1, encoded by *SLC40A1*) for iron export. Iron regulatory proteins (IRP1 and IRP2) are iron sensors to regulate the expression of above iron-related proteins and are regulated by iron. Previous research showed that oxidative stress could induce iron signaling and promote iron uptake [6]. Here, we found that the protein levels of TfR and ferritin were markedly upregulated and that of Fpn1 was downregulated after SAH (Figure 2A), indicating more iron was transported into cells and stored in ferritin, while less iron was released. As a result, cellular iron accumulated. In line with this, the level of IRP2 was decreased (Figure 2A), while IRP1 displayed a similar change to IRP2 due to the elevated ROS level after SAH (see Discussion). Treatment with SC79 significantly reversed the protein levels of iron-related proteins including Fpn1, ferritin, TfR, IRP1, and IRP2 (Figure 2A), indicating rescue of the disrupted iron homeostasis. To further verify the effect of SC79 on iron status of the brain tissue, Perl’s staining and ferrozine assay were performed. The results proved that treatment with SC79 significantly alleviated iron accumulation (Figure 2B,C), suggesting that activation of Akt by SC79 may lessen iron deposition possibly through inhibiting iron uptake and promoting iron export after SAH.

### 2.3. SC79 Administration Rescues the Fe-S Cluster Biogenesis and Fe-S Cluster-Containing Enzymatic Activities and Alleviates Neuronal Injury after SAH

**Figure 3:**
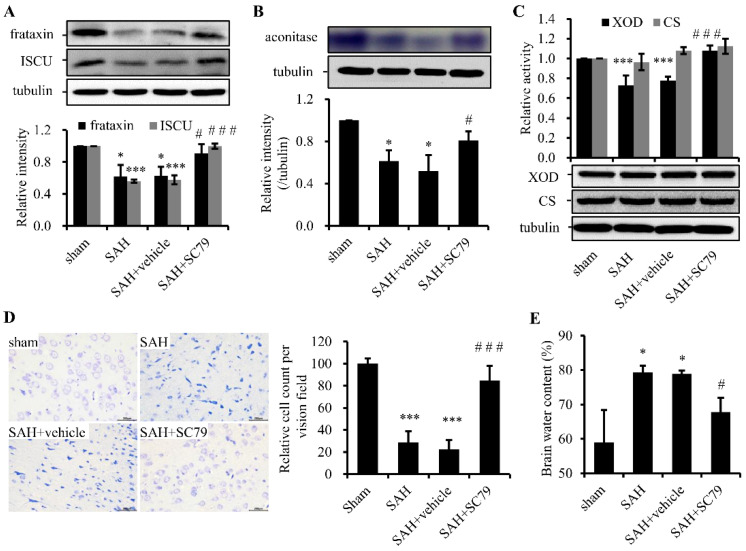
SC79 administration after SAH largely rescues the impairment of Fe-S cluster biogenesis and alleviates the damage of neurons. (**A**) Western blot analysis for frataxin and ISCU. Upper panel: representative protein levels of frataxin and ISCU. Lower panel: quantitative data of the protein levels; (**B**) in-gel assay of aconitase activity. Upper panel: representative result. Lower panel: quantitative data of enzymatic activities; (**C**) enzymatic activity (**upper panel**) and Western blot (**bottom panel**) assays of XOD and CS; (**D**) representative slides of Nissl staining (**left panel**) and quantitative data (**right panel**) visualizing the neuronal cell outline and structure; and (**E**) alterations in brain water content. Data are expressed as mean ± SD (*n* = 8 in each group). * *p* < 0.05, *** *p* < 0.001 vs. sham group, # *p* < 0.05, ### *p* < 0.001 vs. SAH group. ISCU, Fe-S cluster scaffold protein; XOD, xanthine oxidase; CS, citrate synthase.

Fe-S clusters are important cofactor in proteins that are vital for electron transfers, redox reactions, and metabolic catalysis. Either iron accumulation-induced or direct SAH-induced ROS are able to suppress Fe-S cluster biogenesis and disassemble Fe-S clusters. Here we measured the protein levels of ISCU, a major scaffold protein, and frataxin, an iron donor for Fe-S cluster biogenesis, and activities of aconitase and XOD, two Fe-S cluster-containing enzymes, and CS, a non-Fe-S cluster enzyme. Both protein levels of ISCU and frataxin and the activities of aconitase and XOD were notably decreased after SAH compared with the sham group. Treatment with SC79 could significantly prevent the decrease of ISCU and frataxin expression (Figure 3A) and protect the enzymatic activities of aconitase (Figure 3B) and XOD (Figure 3C) compared with that in SAH or SAH + vehicle group. However, the activity and protein levels of CS kept constant in the groups with or without SC79 treatment (Figure 3C). The results indicate that SAH severely and specifically weakened Fe-S cluster biogenesis/assembly and disrupted the function of Fe-S cluster-containing enzymes and that SC79 maintained Fe-S stability to protect the enzymatic activities in the crucial biochemical processes.

As mentioned above, Fe-S clusters are involved in many mitochondrial important metabolic pathways. Deficiency of Fe-S clusters or reduced activities of Fe-S containing enzymes may trigger mitochondrial dysfunction. Neurons are thought to rely on mitochondria more than other types of cells. To further examine whether the rescue of Fe-S cluster biogenesis by SC79 treatment could protect the neurons from injury after SAH, Nissl staining was carried out. For a therapeutic compound to be considered clinically relevant, we provided a delayed window at 4 h after SAH to treat the rats with SC79 in Nissl staining. The data showed that a majority of neurons were severely impaired in the SAH or SAH + vehicle group, exhibiting shrunken cell bodies, condensed nuclei, and dark cytoplasm compared with the sham group (Figure 3D). In contrast, SC79 treatment evidently attenuated the neuronal damage compared with the SAH or SAH + vehicle group. Furthermore, brain water content was determined to be significantly higher in SAH or SAH + vehicle group than in the sham group, whereas it was markedly decreased by SC79 treatment (Figure 3E). The neurological scores were also examined that the vehicle-treated SAH rats showed statistically significant neurological behavior impairment when compared with the sham group. However, when treated with SC79, the rats showed remarkable improvement of neurological defect (not shown), consistent with our another study (revised submission). Taken together, these findings revealed the association between Fe-S cluster biogenesis and Akt activation and suggest that restore of Fe-S cluster biogenesis could be involved in the neuroprotective effect of SC79 in the rat model of SAH.

## 3. Discussion

In this study, we confirm that SC79 may efficiently promote the phosphorylation of Akt and confer neuroprotection after SAH. These neuroprotective effects of SC79 is revealed to be due, at least in part, to its ability to prevent SAH-induced ROS generation and maintain iron homeostasis and Fe-S cluster assembly.

SAH occurs after the rupture of an aneurysm in the cerebral artery wall. Experimental studies have implicated that oxyhemoglobin released from the lysis of red blood cells within the subarachnoid space generates superoxide anion radicals and hydroxyl radicals, which undergo autoxidation to methemoglobin [13]. There is substantial evidence to suggest that the development of cerebral injury after SAH is significantly triggered by oxidative stress, which resulted from disrupted mitochondrial respiration, upregulated free radical producing enzymes, and inhibited intrinsic antioxidant systems [14]. Akt activation could suppress oxidative stress by upregulating SOD1/2 expression [7,8], in agreement with another study (Dingding Zhang, unpublished data) and this study, in which we observed that SC79 decreased ROS production and MDA level through activation of Akt. These studies support a notion for the use of antioxidants in the treatment of aneurismal SAH [14].

Iron is an essential element for all organisms. Iron depletion or repletion were associated with some diseases [15,16]. In the SAH model, red blood cells were lysed within the subarachnoid space. Hemoglobin and its oxidation product, heme, are released. Heme is converted in the brain by heme oxygenase into carbon monoxide, biliverdin, accompanied by the release of catalytically redox-active iron [17], which was transported into cells by the cell surface TfR-mediated iron uptake as transferrin iron. Previous studies have demonstrated that oxidative stress could promote TfR expression for iron uptake [5,6] and inflammation reduce Fpn1 expression to prevent iron release [18]. Consistent with that, here we found that TfR was upregulated, which possibly partially resulted from SAH-induced oxidative stress, and Fpn1 was downregulated, likely from SAH-induced inflammation [19]. SC79 treatment decreased TfR and increased Fpn1 expression to avoid iron accumulation in the brain, which is supported by Perl’s staining and ferrozine assays. Likely, the cyclic enol ether in the structure of SC79 is opened in the presence of iron (Fe^3+^) and the newly-formed beta-two ketone and phenol structure may coordinate with iron. This possible iron chelating activity might contribute to the protective effects as previously reported [4].

The interplay of ROS and excess iron may bring on a vicious outcome, particularly in neurons. Fe-S cluster biogenesis and its incorporation into various Fe-S proteins are extraordinarily sensitive to oxidative stress. Under oxidative stress condition, oxygen species convert Fe-S clusters to unstable forms that quickly decompose [20]. Fe-S clusters directly participate in diverse biological processes, including respiration, DNA replication, and gene regulation [21], and the deficiency of Fe-S clusters will lead to severe alteration of Fe-S enzyme activities [22]. As we observed in this study, SAH-induced ROS does not only retard the Fe-S biogenesis by reduction of the core components, ISCU and frataxin, of Fe-S cluster biogenesis, but also disassembles the integral Fe-S clusters of Fe-S proteins, for instance cytosolic XOD and aconitase IRP1, to reduce the enzymatic activities. When aconitase-IRP1 loses the Fe-S cluster, IRP1 gains an IRE-binding activity to regulate the expression of some iron-related proteins (see next paragraph), one of which is TfR for iron uptake.

IRP1/2 are cytosolic iron-sensing proteins, post-transcriptionally regulating iron-related proteins by the IRP-IRE system [23]. Under low iron circumstances, IRPs bind to IRE in the 5'-untranslated region (UTR) of ferritin and Fpn1 mRNA to inhibit their translation. Similarly, they bind to the IREs in 3′-UTR of TfR mRNA to stabilize the mRNA. Thus, iron uptake is facilitated and iron storage and release are favored [24]. By contrast, when iron is in excess, IRP2 would be unstable and degraded and IRP1 would be converted to aconitase by incorporating a 4Fe-4S cluster [23,24]. The IRE-binding activities of IRPs are diminished. As a result, iron uptake is inhibited and iron storage and release were promoted [24]. However, SAH-induced iron accumulation did not diminish the protein level of TfR and elevate protein level of Fpn1, though both IRP1 and IRP2 protein levels reduced. In addition to the alternative non-IRE mRNA of Fpn1, here, both TfR and Fpn1 are probably not regulated by IRPs; instead, by ROS or inflammation [5,6,18]. IRP2 degradation is iron dependent, whereas IRP1, stabilized by incorporation of Fe-S cluster, is destabilized by ROS despite the excess iron [25], which explained the reduced IRP1 protein level after SAH in this study. SC79 administration after SAH attenuated both oxidative challenge and iron accumulation, consequently, iron homeostasis is then restored. Not only Fe-S cluster biogenesis, but also the activities of Fe-S cluster-containing enzymes were rescued, which maintained mitochondrial function and protected neurons from damage.

Collectively, this study confirmed the beneficial effect of activation of Akt by SC79 administration after SAH. The interplay of oxidative stress and iron accumulation is likely synergistically involved in the severity of the acute phase following SAH. The mechanism of the neuroprotection by SC79 might involve the ability of SC79 to inhibit ROS production and attenuate cellular iron accumulation, further to rescue Fe-S cluster biogenesis and the activities of Fe-S cluster-containing enzymes following SAH. These results support that SC79 might be a potential therapeutic agent for patients suffering from SAH.

## 4. Experimental Section

### 4.1. Animal Preparation

All procedures were approved by the Animal Care and Use Committee of Nanjing University and were conformed to guide for the Care and Use of Laboratory Animals by National Institutes of Health. Male Sprague-Dawley rats (300–350 g) were housed in a humidity-controlled room (25 ± 1 °C, 12 h light/dark cycle) and were raised with free access to water and food.

### 4.2. Prechiasmatic Cistern Blood Injection for SAH Model

Experimental SAH was performed as described previously in our laboratory and other laboratories [26,27]. Briefly, rats were intraperitoneally anesthetized with pentobarbital sodium (50 mg/kg body weight, Jinling hospital, Nanjing, China) and placed in a stereotaxic head frame. An insulin injection needle (BD Science, Franklin Lakes, NJ, USA) was tilted 45° in the sagittal plane and placed 8 mm anterior to the bregma in the midline, with the hole facing the right side. It was lowered until the tip reached the base of the skull, 2–3 mm anterior to the chiasma (approximately 10–12 mm from the brain surface) and retracted 0.5 mm. Loss of cerebrospinal fluid and bleeding from the midline vessels were prevented by plugging the burr hole with bone wax before inserting the needle. A total of 300 µL non-heparinized fresh autologous arterial blood was slowly injected into the prechiasmatic cistern for 3 min under aseptic technique. The heart rate was monitored and the rectal temperature was kept at 37 ± 0.5 °C by using physical cooling (ice bag) when required throughout experiments. Arterial blood samples were analyzed intermittently to maintain pO_2_, pCO_2_, and pH, parameters within normal physiological ranges. To maintain fluid balance, all rats were supplemented with 2 mL of 0.9% NaCl administered subcutaneously. After recovering from anesthesia, rats were returned to their cages with free-access food and water provided adlibitum. The saline control group underwent injection of 300 µL of 0.9% NaCl into the subarachnoid space. Rats that died during surgery or during surgical recovery were excluded, and the procedure was repeated until final group sizes reached the planned experimental number.

### 4.3. Experimental Design and Tissue Preparation

SC79 was purchased from Sigma-Aldrich (St. Louis, MO, USA) and freshly prepared in dimethyl sulfoxide (DMSO) just before intracerbroventricular (ICV) injection. The rat randomly divided into the sham (surgery with normal saline insult, *n* = 8), SAH (*n* = 8), SAH + vehicle (SAH treated with DMSO 5 μL/rat, *n* = 8), SAH + SC79 (SAH treated with SC79 100 μg/rat, *n* = 8) groups. SC79 were injected directly ICV at 30 min after SAH. Then, the rats were sacrificed 24 h after SAH for the biochemical and histological analysis. For Nissl staining, SC79 was injected directly ICV at 4 h after SAH, and sacrificed 72 h after SAH.

Animals were anesthetized as above, and perfused through the left cardiac ventricle with normal saline (4 °C) until effluent from the right atrium was clear. Rats, which had obvious clots in the prechiasmatic cisterns, were selected for further analysis. The temporal lobe tissue, which was near the hematoma, was harvested on ice after blood clots on the tissue were carefully cleared. The fresh tissues were used for measurement of cellular ROS and aconitase activity. The other tissues were stored in −80 °C until further use for Western blotting and ferrozine iron assay. For histological examination, the rats were perfused with normal saline (4 °C) followed by 4% buffered paraformaldehyde (4 °C) and then the brains were immersed in 4% buffered paraformaldehyde (4 °C) for further study.

### 4.4. Iron Perl’s Staining and Ferrozine Iron Assays

Perl’s staining was performed as described previously [28]. Iron content was measured using a colorimetric ferrozine-based assay with some modifications [29]. Briefly, 11 μL concentrated HCl (11.6 mol/L) was added to 50 μL lysate (~250 μg). The mixed sample was heated at 95 °C for 20 min, then centrifuged at 20,000× *g* for 20 min. Supernatant was transferred very gently into fresh tubes. Ascorbate was added to reduce the Fe (III) into Fe (II). Ferrozine and saturate ammonium acetate (NH_4_Ac) were sequentially added to each tube and the absorbance was measured at 570 nm (BioTek EL × 800, Shanghai, China) within 30 min.

### 4.5. Western Blot Analysis

Western blotting was performed as described previously [3]. The information for primary antibodies is as follows: anti-Akt and p-Akt (Ser473) from Cell Signaling Technology (Danvers, MA, USA), anti-ferritin, Fpn1, IRP1, IRP2, and ISCU from Abcam (Cambridge, MA, USA), anti-TfR from Thermo Fisher Scientific Inc. (Waltham, MA, USA), anti-β-tubulin from Sigma-Aldrich, anti-xanthine oxidase (XOD) and citrate synthase (CS) from Proteintech Group, Inc. (Chicago, IL, USA), and anti-frataxin (self-made) [30]. Relative changes of protein expression levels were estimated from the mean pixel density using ImageJ software, normalized to β-tubulin.

### 4.6. Enzymetic Activity Assays

Aconitase activity assays were performed as described previously [3]. Quantitation was performed using ImageJ software. Related chemicals used were purchased from Sigma-Aldrich. Cellular XOD and CS activity were assayed using kits (Nanjing Jiancheng Bioengineering Institute, Nanjing, China) following the manufacturer’s protocols, respectively.

### 4.7. Measurement of Cellular ROS Production and MDA Levels

Cellular ROS production and MDA level were analyzed based on a ROS Detection Kit (Genmed Scientifics Inc., Boston, MA, USA) and a MDA assay kit (Nanjing Jiancheng Bioengineering Institute) following the manufacturer’s protocols, respectively.

### 4.8. Brain Edema

Briefly, after brain tissue was removed and collected, the samples were weighed immediately (wet weight), dried at 100 °C for 48 h, and then weighed (dry weight). The percentage of water content was calculated as [(wet weight − dry weight)/wet weight] × 100%.

### 4.9. Nissl Staining

Formalin-fixed brains (72 h post-SAH) were dehydrated, embedded in paraffin and sliced into four-micrometer thick sections. For Nissl staining, the sections were hydrated in 1% toluidine blue at 50 °C for 20 min. After rinsing with double-distilled water, the sections were dehydrated and mounted with Permount.

### 4.10. Statistical Analysis

All data were expressed as mean ± SD. A one-way analysis of variance (ANOVA) was carried out using SPSS ver. 22.0 software (IBM Corporation, Armonk, NY, USA). Significance was considered at *p* < 0.05.
