# Correction: Hao et al. Phosphorylation of Akt by SC79 Prevents Iron Accumulation and Ameliorates Early Brain Injury in a Model of Experimental Subarachnoid Hemorrhage. *Molecules* 2016, *21*, 325

**DOI:** 10.3390/molecules30040823

**Published:** 2025-02-11

**Authors:** Shuangying Hao, Chuanhui Song, Longcheng Shang, Yu Jiang, Tong Qiao, Kuanyu Li

**Affiliations:** 1 Jiangsu Key Laboratory for Molecular Medicine, Medical School of Nanjing University, Nanjing 210093, China; shuangying9088@163.com (S.H.); songchuanhui1016@hotmail.com (C.S.); sjbslc@126.com (L.S.); 2 Department of Vascular Surgery, the Affiliated Drum Tower Hospital of Nanjing University Medical School, Nanjing 210008, China; rickyjy@163.com

## Author Name Correction

In the published publication [1], there was an error regarding the author name. We changed Jiang Yu to Yu Jiang.

## Error in Figure

In the original publication [1], there was a mistake in Figure 3D as published. There were overlapping panels in Figure 3D’s SAH and SAH+vehicle panels. The corrected Figure 3 appears below.

**Figure 3:**
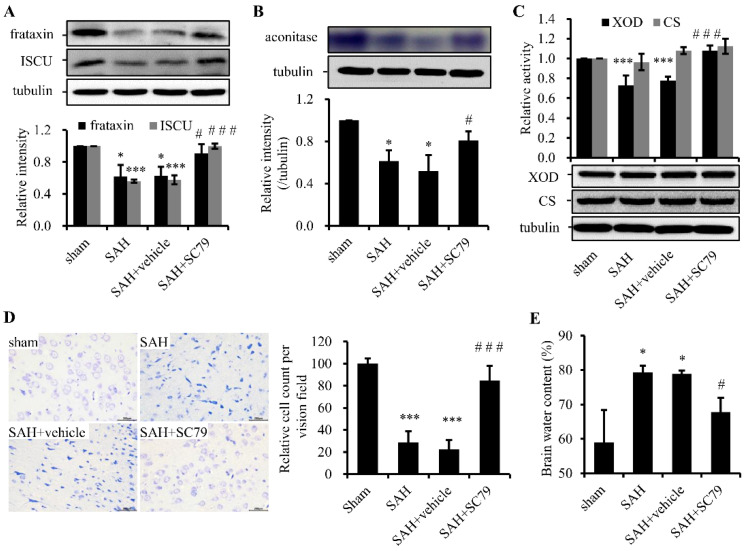
SC79 administration after SAH largely rescues the impairment of Fe-S cluster biogenesis and alleviates the damage of neurons. (**A**) Western blot analysis for frataxin and ISCU. Upper panel: representative protein levels of frataxin and ISCU. Lower panel: quantitative data of the protein levels; (**B**) in-gel assay of aconitase activity. Upper panel: representative result. Lower panel: quantitative data of enzymatic activities; (**C**) enzymatic activity (**upper panel**) and Western blot (**bottom panel**) assays of XOD and CS; (**D**) representative slides of Nissl staining (**left panel**) and quantitative data (**right panel**) visualizing the neuronal cell outline and structure; and (**E**) alterations in brain water content. Data are expressed as mean ± SD (*n* = 8 in each group). * *p* < 0.05, *** *p* < 0.001 vs. sham group, # *p* < 0.05, ### *p* < 0.001 vs. SAH group. ISCU, Fe-S cluster scaffold protein; XOD, xanthine oxidase; CS, citrate synthase.

The authors state that the scientific conclusions are unaffected. This correction was approved by the Academic Editor. The original publication has also been updated.
